# Percutaneous Vegetectomy for Infective Endocarditis in a Nonsurgical Candidate

**DOI:** 10.1016/j.jaccas.2025.105237

**Published:** 2025-09-05

**Authors:** Giovanni Zlaket, Boris Yushuvayev, Paul Gilbert, Samier Deen, Cece Ibeson, Seema Jain, Azeen Elahi, Danielle Stearns, Joshua Waggoner, Kara Asbury

**Affiliations:** HonorHealth Thompson Peak Medical Center, Scottsdale, Arizona, USA

**Keywords:** murmur, right-sided infective endocarditis, tricuspid valve

## Abstract

**Background:**

Surgical management is recommended for infective endocarditis (IE) when there is right heart failure due to severe tricuspid regurgitation, recurrent septic pulmonary emboli, persistent bacteremia, and large tricuspid valve vegetations (≥20 mm). However, sternotomy comes with strict eligibility limitations, including poor functional status, respiratory failure, and recent intravenous drug use.

**Case Summary:**

A 55-year-old woman with a history of intravenous drug use was diagnosed with persistent bacteremia in the setting of tricuspid valve endocarditis. Poor surgical candidacy prompted consideration of less invasive alternatives, including percutaneous aspiration.

**Discussion:**

This case highlights the viability of AngioVac vegetectomy as an alternative treatment modality for persistent IE in poor surgical candidates.

**Take-Home Messages:**

Surgical intervention is considered for right-sided native valve IE with vegetations ≥20 mm, persistent bacteremia, recurrent pulmonary septic emboli, or highly resistant organisms. The AngioVac system provides a minimally invasive treatment strategy for IE in patients who are otherwise ineligible for surgical intervention.

## Graphical Abstract

**Figure:**
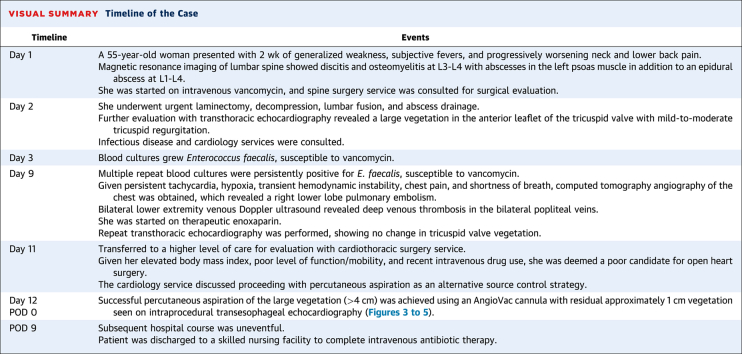

## History of Presentation

A 55-year-old woman presented with 2 weeks of generalized weakness, subjective fevers, and progressively worsening neck and lower back pain. On presentation, she was afebrile and hemodynamically stable. Initial physical examination revealed a grade III/VI holosystolic murmur, worse on inspiration, loudest at the apex, and a gallop loudest at the left sternal border. Motor strength assessment revealed reduced 4/5 strength in the left upper and lower extremities.

**Take-Home Messages**

• Surgical intervention is considered for right-sided native valve IE with vegetations ≥20 mm, persistent bacteremia, recurrent pulmonary septic emboli, or highly resistant organisms.
• The AngioVac system provides a minimally invasive treatment strategy for IE in patients who are otherwise ineligible for surgical intervention.

## Past Medical History

The patient's past medical history was significant for morbid obesity (body mass index 42 kg/m^2^) and well-controlled asthma. On further questioning, she admitted to recent intravenous methamphetamine use.

## Differential Diagnosis

Differential diagnosis based on initial presentation included infective endocarditis (IE) with associated epidural abscess or vertebral osteomyelitis, acute valvulopathy with associated musculoskeletal pain secondary to acute rheumatic fever, spinal cord infarction resulting from emboli secondary to IE, and carcinoid heart disease with metastasis to the spine.

## Investigations

**Figure 1:**
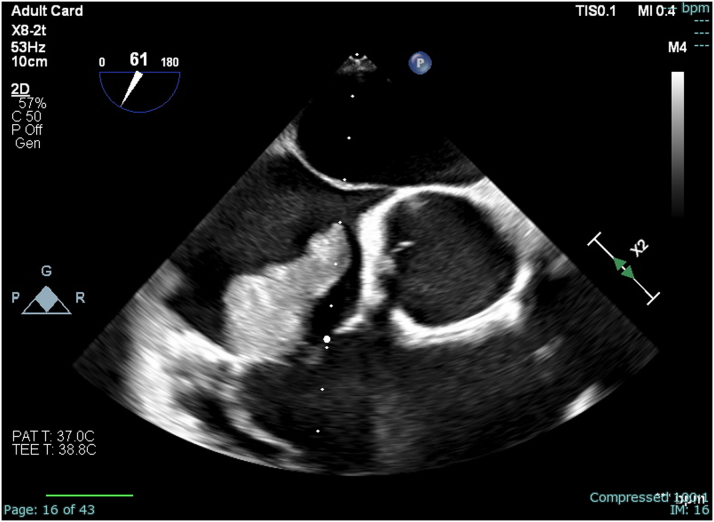
Large Vegetation (>3 cm) Present on the Tricuspid Valve on Midesophageal Right Ventricular Inflow-Outflow Preprocedural Transesophageal Echocardiography

Initial investigative work-up with magnetic resonance imaging of the lumbar spine showed discitis and osteomyelitis at L3-L4 with abscesses in the left psoas muscle in addition to an epidural abscess at L1-L4. Further evaluation with transthoracic echocardiography revealed a large vegetation in the anterior leaflet of the tricuspid valve (TV) with mild-to-moderate tricuspid regurgitation (Figure 1).

## Management (Medical/Interventions)

Concerning her lumbar spine osteomyelitis complicated by abscesses, the patient underwent urgent laminectomy, decompression, lumbar fusion, abscess drainage and was initiated on intravenous vancomycin. Blood cultures grew *Enterococcus faecalis*, and despite appropriate antibiotic therapy, both bacteremia and TV vegetation persisted for 8 days, prompting evaluation for surgical resection. She was transferred to a higher level of care for cardiothoracic surgery evaluation. However, given her elevated body mass index, poor level of function, and recent intravenous drug use, she was deemed not an appropriate surgical candidate.

## Outcome and Follow-Up

**Figure 2:**
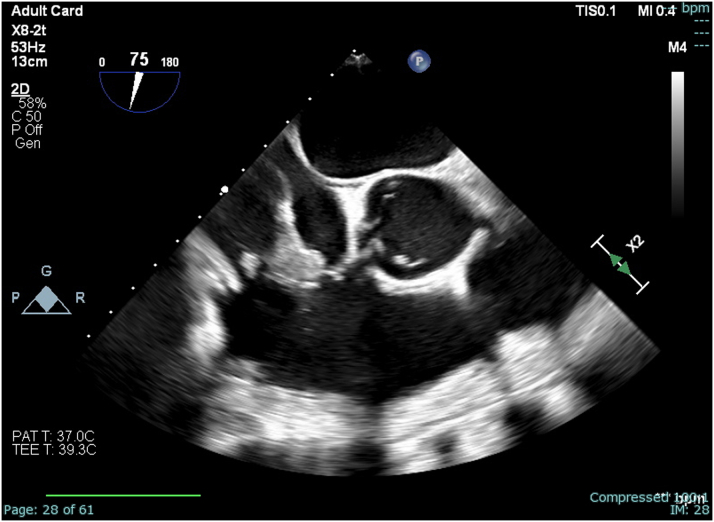
Midesophageal Right Ventricular Inflow-Outflow View During Procedural Transesophageal Echocardiography Thrombectomy catheter is engaged with thrombus midaspiration.

**Figure 3:**
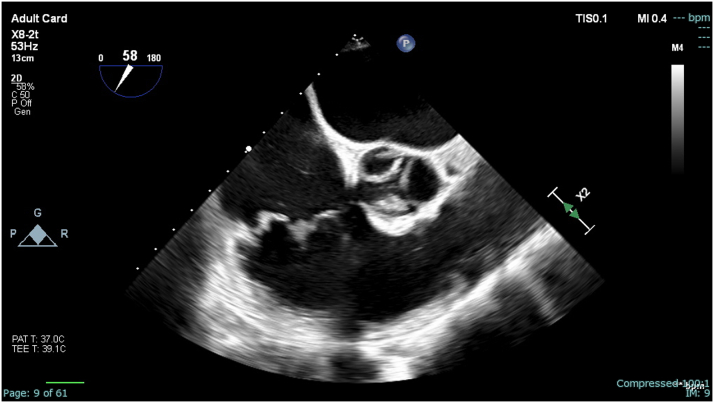
Residual Vegetation (approximately 1 cm) Present on the Tricuspid Valve on MidEsophageal Right Ventricular Inflow-Outflow Post-Thrombectomy

**Figure 4:**
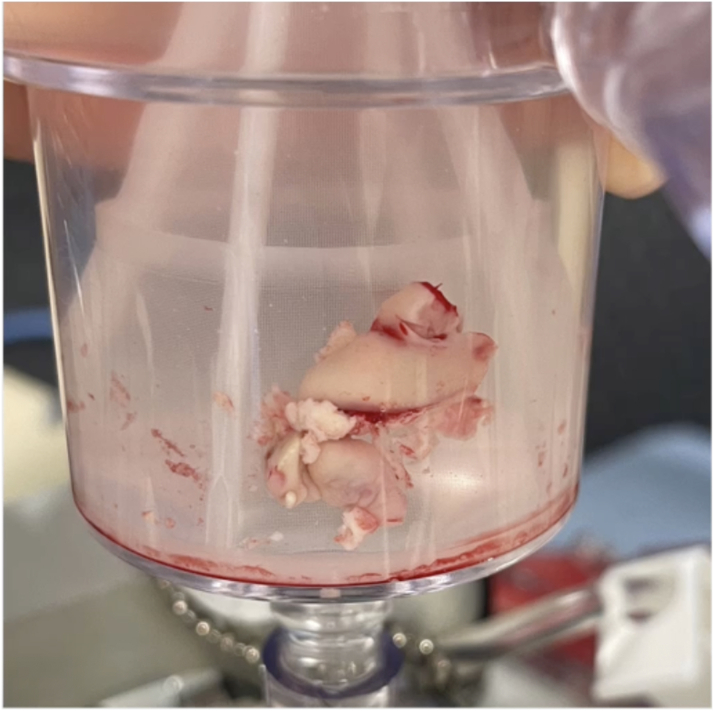
Large Vegetation (>4 cm) Extracted Using the AngioVac Cannula System

**Figure 5:**
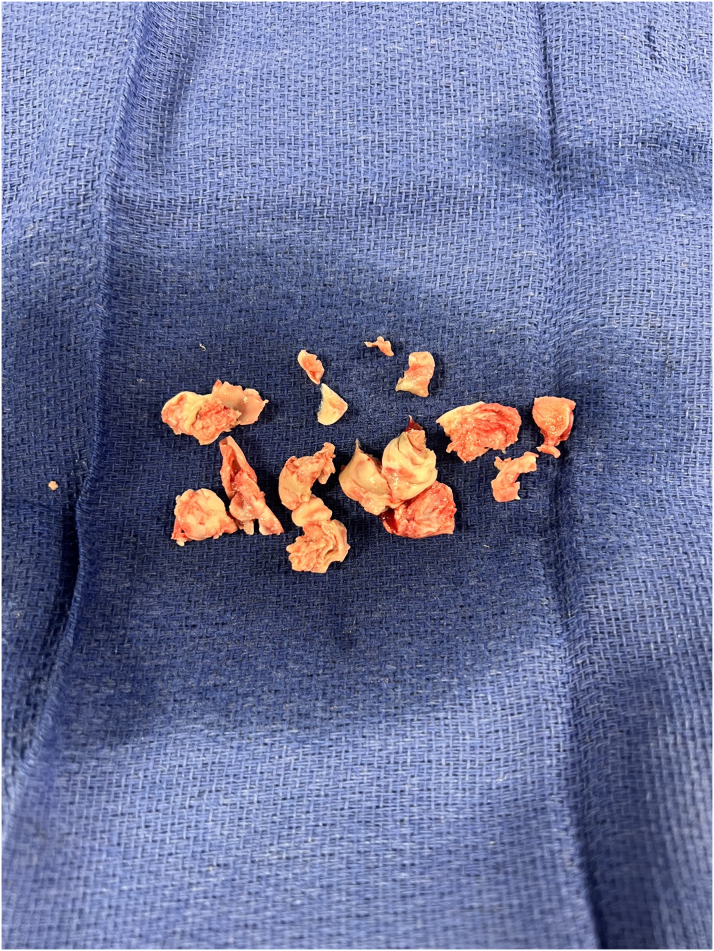
Large Vegetation (>4 cm) Shown on a Surgical Specimen Tray

Hospitalization was complicated by recurrent septic pulmonary emboli, which further prompted the need for percutaneous intervention. Therefore, the patient underwent aspiration of the large vegetation (>4 cm) using an AngioVac cannula (AngioDynamics, Inc), a percutaneous mechanical suction device, with residual approximately 1 cm vegetation seen on intraprocedural transesophageal echocardiography (Figures 2, 3, 4, and 5). During the procedure, both right and left common femoral veins (CFVs) were accessed via 8-F sheaths. A 26-F DrySeal Flex (W. L. Gore & Associates, Inc) sheath was then inserted via exchange in the right CFV through which the AngioVac suction catheter was introduced, advanced to the right atrium, and positioned over the TV vegetation via transesophageal echocardiography guidance. The circuit was then connected to an extracorporeal tubing system, which allowed for blood filtering before its reinfusion through the left CFV. After several suctioning attempts, the majority of the TV vegetation was successfully evacuated without complications. She remained hemodynamically stable during the procedure. The hospital course was uneventful thereafter, and she was discharged to a skilled nursing facility to complete intravenous antibiotic therapy.

## Discussion

Current evidence supports surgical management strategies for IE when there is right heart failure due to severe tricuspid regurgitation, recurrent septic pulmonary emboli, persistent bacteremia, and large TV vegetations (≥20 mm).^1^^,^^2^ Surgical debridement with cardiopulmonary bypass is considered a primary treatment approach for right-sided IE where source control is required.^3^ However, patient candidacy restrictions constitute a major barrier to care for an otherwise fatal disease process if inadequately treated.^1^, ^2^, ^3^ This case highlights the viability of AngioVac vegetectomy as an efficacious alternative treatment modality for persistent IE despite appropriate antimicrobial therapy in a poor surgical candidate. Further research should be conducted to characterize the role, safety, and efficacy of percutaneous intervention in IE.

## Conclusions

AngioVac-assisted vegetation debulking is a viable therapeutic strategy for treatment-refractory right-sided IE in patients with elevated perioperative risk.^2^^,^^4^

**Visual Summary:** Timeline of the Case

Timeline	Events
Day 1	A 55-year-old woman presented with 2 wk of generalized weakness, subjective fevers, and progressively worsening neck and lower back pain. Magnetic resonance imaging of lumbar spine showed discitis and osteomyelitis at L3-L4 with abscesses in the left psoas muscle in addition to an epidural abscess at L1-L4. She was started on intravenous vancomycin, and spine surgery service was consulted for surgical evaluation.
Day 2	She underwent urgent laminectomy, decompression, lumbar fusion, and abscess drainage. Further evaluation with transthoracic echocardiography revealed a large vegetation in the anterior leaflet of the tricuspid valve with mild-to-moderate tricuspid regurgitation. Infectious disease and cardiology services were consulted.
Day 3	Blood cultures grew *Enterococcus faecalis*, susceptible to vancomycin.
Day 9	Multiple repeat blood cultures were persistently positive for *E. faecalis*, susceptible to vancomycin. Given persistent tachycardia, hypoxia, transient hemodynamic instability, chest pain, and shortness of breath, computed tomography angiography of the chest was obtained, which revealed a right lower lobe pulmonary embolism. Bilateral lower extremity venous Doppler ultrasound revealed deep venous thrombosis in the bilateral popliteal veins. She was started on therapeutic enoxaparin. Repeat transthoracic echocardiography was performed, showing no change in tricuspid valve vegetation.
Day 11	Transferred to a higher level of care for evaluation with cardiothoracic surgery service. Given her elevated body mass index, poor level of function/mobility, and recent intravenous drug use, she was deemed a poor candidate for open heart surgery. The cardiology service discussed proceeding with percutaneous aspiration as an alternative source control strategy.
Day 12 POD 0	Successful percutaneous aspiration of the large vegetation (>4 cm) was achieved using an AngioVac cannula with residual approximately 1 cm vegetation seen on intraprocedural transesophageal echocardiography (Figures 3, 4, and 5).
POD 9	Subsequent hospital course was uneventful. Patient was discharged to a skilled nursing facility to complete intravenous antibiotic therapy.

## Funding Support and Author Disclosures

The authors have reported that they have no relationships relevant to the contents of this paper to disclose.
